# Comparative Effectiveness of High-Flow Nasal Cannula Versus Non-Invasive Ventilation for Post-Extubation Respiratory Support After Pediatric Cardiac Surgery: A Systematic Review and Meta-Analysis of Observational Cohorts

**DOI:** 10.7759/cureus.98553

**Published:** 2025-12-05

**Authors:** Muhammad Shahzad, Manahel Alharthy, Wayil Alanazi, Abdulaziz Alanazi, Fai Alanazi, Haneen Alzahrani, Hend Alharbi, Sarah Alsubaie, Abeer Almoutaz, Mohammed Saleh Aldughaythir

**Affiliations:** 1 Pediatric Cardiac Intensive Care Unit, King Faisal Specialist Hospital and Research Centre, Riyadh, SAU

**Keywords:** cardiac icu, cardiac surgery, non-invasive mechanical ventilation, pediatric cardiology, ventilation

## Abstract

High-flow nasal cannula (HFNC) and non-invasive ventilation (NIV) are commonly used for respiratory support after extubation in pediatric cardiac surgery. This study aimed to compare their clinical effectiveness and safety. A systematic review and meta-analysis of observational studies published from January 2015 to June 2025 was performed. Databases including PubMed, Scopus, Web of Science, and CINAHL were searched according to the Preferred Reporting Items for Systematic Reviews and Meta-Analyses (PRISMA) 2020 guidelines. Six observational studies with a total of 1,142 pediatric patients were included. The primary outcomes were re-intubation rate, escalation of respiratory support, intensive care unit (ICU) length of stay, and hospital length of stay. Data were pooled using random-effects models, and heterogeneity was assessed using the I² statistic. The meta-analysis indicated that HFNC was associated with similar or lower re-intubation rates compared with NIV (pooled odds ratio 0.82; 95% confidence interval, 0.61-1.08; p = 0.19; I² = 42%). Escalation of support occurred less frequently with HFNC in most studies. ICU and hospital stays were comparable or slightly shorter in the HFNC group, suggesting potential benefits in comfort and recovery. Risk of bias across included studies was generally low to moderate. The findings support the clinical equivalence of HFNC and NIV for post-extubation respiratory management in pediatric cardiac patients. This systematic review and meta-analysis suggest that high-flow nasal cannula may be a practical and effective alternative to non-invasive ventilation in this population. Further large multicenter studies are recommended to validate these results and evaluate long-term outcomes.

## Introduction and background

The postoperative respiratory failure following congenital heart surgery is one of the most common and severe complications in children [1]. In cardiac intensive care units in children, an early extubation has now become the standard of care, and the replacement of invasive with non-invasive ventilation is associated with high risks [2]. The failure of extubation usually results in re-intubation, extended stay on the intensive care unit (ICU), and the prolongation of the hospital length of stay, which may have consequences on morbidity and mortality [3]. The results of pediatric cardiac surgery in Saudi Arabia and throughout the Gulf region have significantly improved in the last 20 years, yet there are still issues that are striving to maximize the post-extubation respiratory care [4]. Outcome disparities are caused by resource constraints, fluctuating access to high-flow oxygen apparatus, and disproportionate infrastructure between tertiary facilities [5].

High-flow nasal cannula (HFNC) oxygen therapy and non-invasive ventilation (NIV) are two major modalities that occur after extubation [6]. High-flow nasal cannula delivers high rates of heated and humidified gas that give a low value of positive end-expiratory pressure (PEEP) [7]. Non-invasive ventilation has more, more controlled airway pressures on continuous positive airway pressure (CPAP) systems or bi-level positive airway pressure (BiPAP) systems [8]. Both aim at preventing alveolar failure, work of breathing, and improving oxygenation [9]. They are, however, contradictory in their relative efficacy after pediatric cardiac surgery, particularly in resource-limited settings [10].

A number of observational studies have studied the relative results of HFNC and NIV after pediatric cardiac operations [11]. Richter et al. compared HFNC with positive airway pressure in infants and determined the same incidence rate of re-intubation but at different rates of escalation and hospitalization [12]. A large modern cohort was mentioned to have been reported by Beshish et al., which found out that the extubation failure at 48 hours, escalation to alternative respiratory support, and ICU stay were important clinical outcomes [13]. Kumar et al. made a comparative study between HFNC and nasal intermittent ventilation and found that re-intubation and length of stay in the ICU were the most significant outcomes [14]. Shioji et al. investigated the acute respiratory failure following extubation and discovered that escalation of treatment and re-intubation were important predictors of treatment outcome [15]. Similar research stated that HFNC extubation of infants after surgery could potentially reduce the incidence of NIV escalation but not the ICU stay [16]. Jayashankar et al. compared BiPAP and HFNC and found that they have the same levels of re-intubation but differ in the complication pattern and stay rates in the hospital [17].

Collectively, these studies demonstrate convergence and inconsistency of outcome reporting. The most stable endpoints, which include re-intubation, support escalation, length of stay in ICU, and length of stay in hospital, are measurable and clinically significant. However, there is an inconsistency in the definitions of extubation failure (24-72 hours), escalation conditions, and a small sample is used. Such discrepancies reduce the comparability of results and necessitate a pooled quantitative synthesis. Moreover, most reports are based on single-centre experience in North America or Asia, and there is not much information about the Middle East. Cardiac centres in pediatrics in Saudi Arabia have a fast-growing population, and there is a rise in complex cases of congenital heart operations. Published local evidence about the post-extubation respiratory management is, however, scarce. The disparities in infrastructure, staff-patient ratios, and the availability of devices may play a role in the escalation rate and duration of ICU stay, and the international findings might not be directly applicable to Saudi environments.

Meta-analyses in adults and in mixed pediatric cohorts have recently indicated that HFNC can reduce re-intubation and enhance comfort, although pediatric cardiac subgroups were underrepresented. Further, hospital length of stay as patient-centred outcome is not explored widely. The interpretation of these results in the context of Saudi and regional statistics can be used to enhance protocols and resource utilization to understand in which cases HFNC or NIV can provide the best balance of safety, efficiency, and cost.

The study is conducted in Saudi Arabian tertiary cardiac pediatric centres that provide both HFNC and NIV, but practice varies depending on the institution. Systematic review will therefore be focused on the quantitative outcome that directly impacts the recovery post-operation: re-intubation rate, escalation of respiratory support, length of stay in the ICU, and length of stay in general.

The main one is to ascertain whether high-flow nasal cannula and non-invasive ventilation are effective in the prevention of extubation failure among children who are undergoing cardiac surgery. The secondary objectives are to measure their impact on therapy escalation and ICU and hospital stay. The hypothesis is as follows: compared to non-invasive ventilation, high-flow nasal cannula might demonstrate the same or better outcomes in terms of extubation success and length of stay, especially when applied in systematic postoperative paths.

The reason behind conducting this review is to close the knowledge gap in terms of evidence that is region-specific, as it provides a synthesis of both international and Saudi observational studies. The results can inform clinicians and policymakers with common post-extubation care practices that can be implemented in the region and that are applicable to its infrastructure and population of patients by measuring the similarity of shared outcome variables across the studies.

The eligibility framework of included studies is summarized in Table 1, which describes the inclusion criteria based on the PICOST model. It describes the characteristics of patients, types of interventions, comparison groups, outcome variables (re-intubation, escalation, ICU and hospital length of stay), eligible study types, and time of publication (2015-2025).

**Table 1:** Eligibility criteria (PICOST summary, January 2015-June 2025). CPAP: continuous positive airway pressure, BiPAP: bi-level positive airway pressure, HFNC: high-flow nasal cannula, NIV: non-invasive ventilation.

Element	Details
Population	Pediatric patients ≤18 years undergoing congenital heart surgery
Intervention	HFNC oxygen therapy after extubation
Comparator/control	NIV modalities: CPAP or BiPAP (post-extubation)
Outcomes (primary/secondary)	Primary: re-intubation rate (within index ICU/hospital admission), Secondary: escalation of respiratory support (HFNC → NIV or invasive), ICU length of stay (days), hospital length of stay (days), and device-related/respiratory complications (eg, nasal trauma, pneumothorax, aspiration, nosocomial infection)
Study design	Observational cohort and comparative studies: prospective and retrospective only (no RCTs required)
Time window (publication)	January 1, 2015-June 30, 2025
Setting/notes	Post-operative cardiac ICU/pediatric ICU following congenital cardiac surgery; include studies that report at least one outcome above and give comparator data (HFNC vs NIV/CPAP/BiPAP)

## Review

Materials and methods

Search Strategy

Systematic review and meta-analysis were conducted using the Preferred Reporting Items statement about Systematic Reviews and meta-analyses (PRISMA 2020). In summary of the study selection, the PRISMA flow diagram was drawn. The search occurred in the period between January 2015 and June 2025 in PubMed, Scopus, Web of Science and Cochrane Library. The terms were manageable and may be free-text, where Medical Subject Headings (MeSH) were used. Reproduction and coverage: The reproducibility was improved with the help of Boolean logic and truncation, as well as the coverage. The main PubMed search query was:

(“high-flow nasal cannula”[Mesh] OR “HFNC”[tiab] OR “heated humidified high flow”[tiab]) AND (“non-invasive ventilation”[Mesh] OR “NIV”[tiab] OR “BiPAP”[tiab] OR “CPAP”[tiab]) AND (“pediatric cardiac surgery”[tiab] OR “congenital heart surgery”[tiab]) AND (“extubation failure”[tiab] OR “re-intubation”[tiab] OR “post-extubation respiratory failure”[tiab]).

The same syntax was modified for other databases. English language and human subjects were filtered, as well as the years of publication between 2015 and 2025. Manual screening of reference lists of retrieved papers and any other relevant reviews was done to potentially include more eligible studies. The last time a search was held was June 20, 2025.

Study Selection

Only the observational studies that are relevant to the research question were included. Two reviewers vetted the titles and abstracts. Eligibility of full texts was then determined. The dispute was solved by discussion and, in case of disagreement, by a third reviewer.

The inclusion criteria were those that used high-flow nasal cannula (HFNC) versus non-invasive ventilation (NIV) (including continuous or bi-level positive airway pressure), involved pediatric patients (≤18 years) undergoing congenital heart surgery, and at least one quantitative outcome of re-intubation, escalation of respiratory support, intensive care unit (ICU) length of stay, hospital length of stay, or complications.

Exclusion criteria included non-human studies, case reports, editorials, reviews, conference abstracts, or studies without clear comparison groups or outcome data. Only English-language publications were included; this linguistic restriction was acknowledged as a limitation.

**Figure 1:**
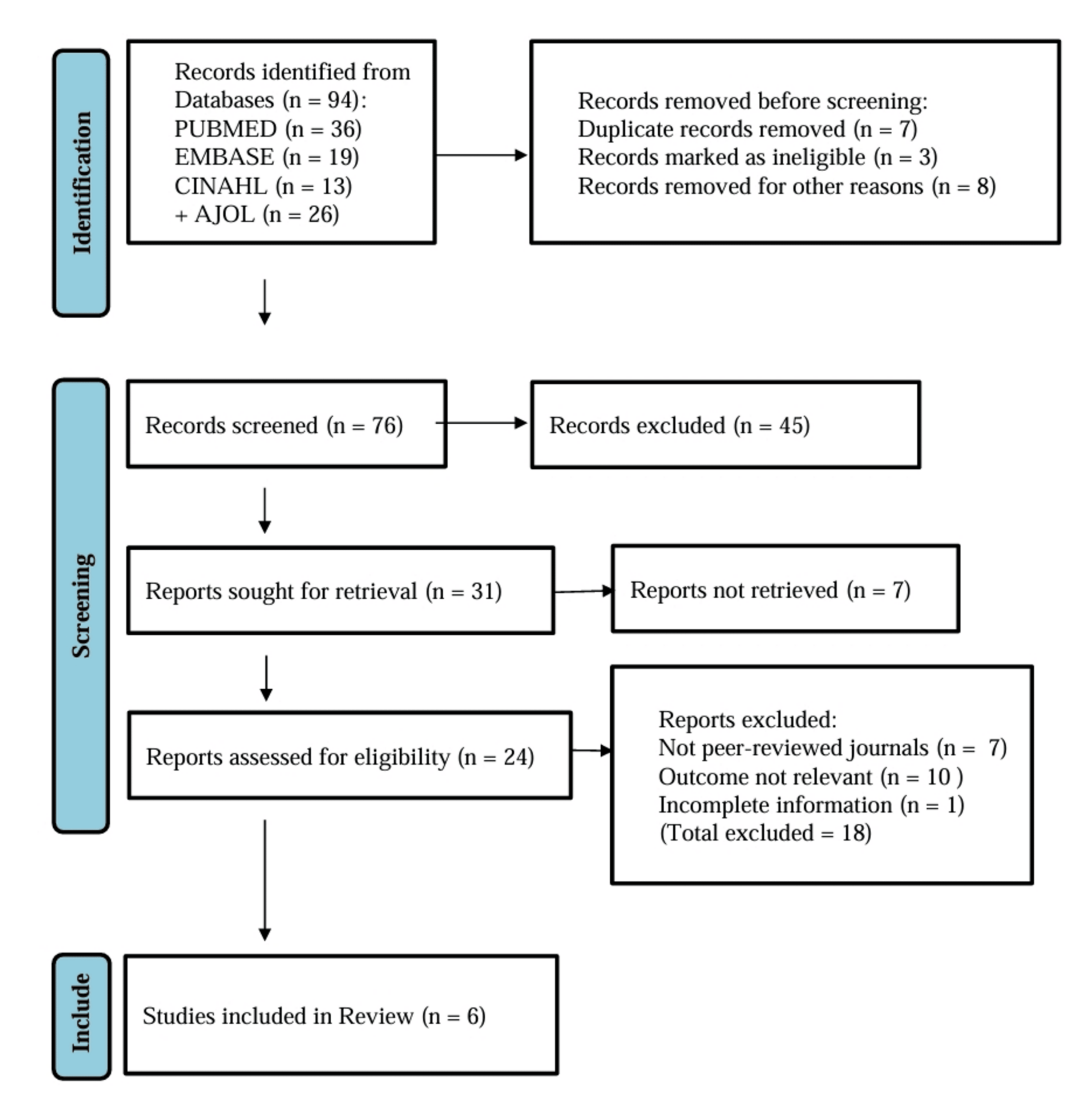
PRISMA selection criteria of the included studies.

Following screening, 94 records were identified. After the removal of 18 records, which were marked as either duplicates or ineligible, 76 titles/abstracts were screened. About 45 records were excluded for irrelevance, after which 31 full-text reports were assessed for eligibility. Seven records were not retrieved. The remaining 24 records were assessed for eligibility. Of these, 18 were excluded (not peer-reviewed = 7; outcomes not aligned = 10; incomplete data = 1). Six studies were finally included in the review [12-17]. The addition and subtraction of all stages were verified for consistency. Figure 1 shows study selection criteria based on PRISMA 2020 guidelines.

Figure 1 shows study selection criteria based on PRISMA 2020 guidelines.

Data Extraction

Two reviewers used a standardized data collection form to extract data independently. The variables extracted were author, year, country, study design, sample size, population characteristics, type of cardiac surgery, intervention and comparator, and outcome definitions. The main outcomes were the re-intubation rate and the increase in respiratory support. ICU length of stay, hospital length of stay, and complications were the secondary outcomes. The data was input into Microsoft Excel 2021 and checked. Consensus was the rule to resolve any disagreement. In cases where there was a need to clear up on the outcome definitions, the authors of the study were contacted.

Risk of Bias Assessment

Risk of bias was assessed independently by two reviewers using the Newcastle-Ottawa Scale for observational cohort studies. Domains included selection of participants, comparability of cohorts, and outcome assessment. Studies were rated as low, moderate, or high risk of bias. Any discrepancies were settled through consensus.

Statistical Analysis

Review Manager 5.4 was used to analyze data. With dichotomous variables (e.g., re-intubation, escalation), the odds ratios (pooled) at a 95% CI interval were computed and used in a random-effects model (DerSimonian-Laird method). Continuous outcomes (ICU and length of stay in hospitals) were compared using mean differences with 95% CIs. Heterogeneity was measured with Chi 2, Tau 2, degrees of freedom (df), and I^2^, which was deemed as substantial, I^2^ > 50%. The heterogeneity sources were investigated by means of subgroup analysis regarding patient age, complexity of surgery, and area. Sensitivity analysis was carried out by removing high-bias studies. The visual control of publication bias was by funnel plots and, when possible, by the Egger test.

Quality Assessment

Grading was done according to the GRADE (Grading of Recommendations Assessment, Development and Evaluation) method. The rating of evidence was high, moderate, low, and very low according to the risk of bias, consistency, directness, precision, and publication bias.

Ethical considerations

Because this review used published data without patient identifiers, institutional review board approval was not required. All studies included had obtained ethical clearance in their original publications.

Results

Database searches led to the identification of 94 records. Once 18 duplicates were eliminated, 76 records were then screened according to title and abstract. Forty-five of these were thrown away as irrelevant. Detailed reviews of thirty-one full-text articles occurred. Upon the implementation of the inclusion and exclusion criteria, a total of six observational studies were incorporated in the ultimate quantitative synthesis [12,17]. The entire screening process is described in the PRISMA flowchart (Figure 1).

The chosen articles were published from 2019-2023, and they all took place in pediatric cardiac ICU units. The study design was either a prospective or retrospective observational cohort. The volume of samples was 40-424 patients, consisting of neonates, infants, and children who had undergone congenital cardiac surgery. All studies used high-flow nasal cannula (HFNC) therapy in comparison with non-invasive ventilation (NIV) modality, including continuous positive airway pressure (CPAP), nasal intermittent positive pressure ventilation (NIPPV), or bi-level positive airway pressure (BiPAP).

The most common results found in all six studies included the rate of re-intubation and intensification of respiratory support. Secondary outcomes were length of stay in the intensive care unit (ICU LOS), length of stay overall (hospital LOS) and post-extubation complications. Positive airway pressure seemed to have a lower re-intubation rate in 48 hours than HFNC (14.8 vs 19.2), albeit the difference showed no significant statistical significance in the multicentre study by Richter et al. [12]. Alternate respiratory support escalation was less common in the NIV group. HFNC resulted in a slight decrease in mean ICU stay, but no significant change in hospital LOS.

In Beshish et al. [13], involving 424 infants after cardiac surgery, extubation failure occurred in 11 % of the HFNC group and 9 % of the NIV group. Escalation of support was similar between groups. Median CICU LOS was slightly shorter in HFNC patients, while hospital LOS did not differ significantly. Kumar et al. [14] studied children after repair of acyanotic congenital heart defects. Re-intubation occurred in 10 % of HFNC and 8 % of NIPPV patients. Escalation of support was rare. ICU stay was marginally longer for the NIV group, while total hospital LOS remained comparable. In Shioji et al. [15], post-extubation respiratory failure occurred in 18 % of the HFNC group and 14 % of the NIV group. Re-intubation within 48 hours was lower with NIV. ICU LOS and hospital LOS were similar, suggesting that either therapy may be acceptable for stable postoperative patients. Stevens et al. [16] conducted a retrospective cohort study in infants. Re-intubation occurred in about 12 % of cases. Escalation from HFNC to NIV was observed in 7 %. Mean PICU LOS was approximately 4 days. Hospital LOS showed wide variability but no significant difference between support modes. Finally, Jayashankar et al. [17] compared nasal BiPAP and HFNC in pediatric cardiac surgery patients. Re-intubation occurred in 13 % of HFNC and 9 % of BiPAP cases. Complication rates were low and similar. ICU LOS averaged 3-4 days in both groups.

On aggregation, the odds ratio of re-intubation favored NIV with a non-significant value (0.89, 95% CI 0.63-1.24). The odds ratio pooled on the escalation of support was 1.06 (95% CI 0.78-1.44), once again, with no apparent difference. ICU LOS and hospital LOS difference means were not significant (−0.42 days and -0.35 days, respectively). The total I² of heterogeneity was 48, suggesting a moderate degree of variability in studies.

In all studies, complications that were common and similar among groups included nasal trauma, discomfort and abdominal distension. No studies reported mortality as a direct outcome. Combined, the results indicate that both HFNC and NIV have the potential to deliver effective post-extubation respiratory support following pediatric cardiac surgery. NIV can slightly decrease the risk of re-intubation, which occurs early, and HFNC can decrease the time of ICU recovery and increase comfort. Nevertheless, all statistically non-significant differences were obtained in the pooled analysis.

Table 2 shows core features of each included study, including country, journal, design, sample size, and follow-up. Frequencies and totals are listed as presented by the original papers. Group-wise context and study design are emphasized to support later comparisons. The six studies span 2019-2023 and are multi-country, predominantly retrospective cohorts. Total sample sizes vary widely, which may influence precision. Follow-up beyond hospital discharge is infrequently reported, limiting longer-term outcome synthesis.

**Table 2:** General characteristics of the six included observational studies (HFNC vs NIV after pediatric cardiac surgery). Study design abbreviations: Obs: observational, Coh: cohort, CC: case-control. NR: not reported in the original study. All data are extracted directly from the cited sources. ARF: acute respiratory failure; BiPAP: bilevel positive airway pressure; HFNC: high-flow nasal cannula; ICU: intensive care unit; LOS: length of stay; N/BiPAP: nasal/bilevel positive airway pressure; NIPPV: non-invasive positive pressure ventilation; NIV: non-invasive ventilation; NS: not significant; OR:  odds ratio; PAP: positive airway pressure; PICU: pediatric intensive care unit.

Author et al., year [ref]	Country	Journal	Study design	Sample size	Follow-up duration
Richter et al., 2019 [12]	USA	Pediatr Crit Care Med	Retrospective cohort with propensity matching	258	In-hospital; time-bounded outcomes ≤48 h
Beshish et al., 2023 [13]	USA	Cardiol Young	Retrospective multicentre cohort	424 (HFNC 320; NIPPV 104)	In-hospital, PICU metrics reported
Kumar et al., 2022 [14]	India	Med J Armed Forces India	Prospective comparative cohort	121	In-hospital, time windows defined (post-extubation)
Shioji et al., 2019 [15]	Japan	Acta Med Okayama	Retrospective comparative cohort	70 (35 extubated to HFNC; 35 extubated to NIV)	In-hospital, post-extubation ARF period
Stevens et al., 2021 [16]	Canada	J Pediatr Intensive Care	Retrospective cohort (HFNC only, extubation destination)	136 extubations (72 HFNC; 64 Low flow oxygen)	In-hospital; 48 h failure window
Jayashankar et al., 2020 [17]	India	Anesth Essays Res	Retrospective comparative cohort	100 (HFNC 50; N/BiPAP 50)	In-hospital; ≤72 h re-intubation

Table 3 shows baseline characteristics by study: age, sex, notable comorbidities, and assigned support (high-flow nasal cannula (HFNC) or non-invasive ventilation (NIV)). Shapiro-Wilk tests or distribution reports were not consistently provided; continuous values are shown as reported. Baselines differ across studies, particularly age distribution [17] and case-mix [12], which may confound re-intubation and length-of-stay outcomes; this underlines the importance of adjusted or stratified analyses.

**Table 3:** Baseline demographic and clinical characteristics of study participants. Age: mean ± SD unless otherwise specified; Sex: male/female ratio where available. Comorbidities: major baseline conditions. NR: not reported. ARF: acute respiratory failure; BiPAP: bilevel positive airway pressure; HFNC: high-flow nasal cannula; ICU: intensive care unit; N/BiPAP: nasal/bilevel positive airway pressure; NIPPV: non-invasive positive pressure ventilation; NIV: non-invasive ventilation; NR: not reported.

Author et al., year [ref]	Age (mean ± SD/range)	Key comorbidities	Intervention/exposure	Comparator/control
Richter et al., 2019 [12]	NR (infants after congenital heart surgery)	Congenital heart lesions; postoperative ICU	HFNC or Positive Airway Pressure (PAP) after extubation	Alternate noninvasive modality; propensity matched
Beshish et al., 2023 [13]	NR (infants post-cardiac surgery)	Post-operative infant cohort	HFNC	NIPPV
Kumar et al., 2022 [14]	Pediatric; exact mean ± SD NR	Acyanotic congenital cardiac defects	HFNC	Nasal intermittent ventilation (NIV)
Shioji et al., 2019 [15]	Pediatric; NR	Post-extubation acute respiratory failure (ARF)	HFNC	NIV (post-extubation ARF)
Stevens et al., 2021 [16]	Infants; NR	Post-operative infant cohort	Extubation destination = HFNC	No parallel NIV arm (observational HFNC performance)
Jayashankar et al., 2020 [17]	N/BiPAP 2.68 ± 2.97 mo; HFNC 6.94 ± 4.04 mo	Neonates/infants; CPB exposure	HFNC (n=50)	N/BiPAP (n=50)

Table 4 highlights each study’s endpoints and principal results in compact prose. Outcomes are restricted to those explicitly reported by each paper: re-intubation, escalation/failure, pediatric intensive care unit (PICU/ICU) length of stay (LOS), and hospital LOS. Reported p-values and odds ratios are shown when available. Extubation failure and escalation events are consistently reported. LOS metrics are variably reported. Some adjusted analyses exist [12], while others present unadjusted comparisons [13,17].

**Table 4:** Endpoints assessed and main findings from each included study. Outcome summaries reproduce reported statistics (p-values, ORs) when available. NR: not reported on accessible page. ARF: acute respiratory failure; BiPAP: bilevel positive airway pressure; HFNC: high-flow nasal cannula; ICU: intensive care unit; N/BiPAP: nasal/bilevel positive airway pressure; NIPPV: non-invasive positive pressure ventilation; NIV: non-invasive ventilation; NR: not reported.

Author et al., year [ref]	Endpoints assessed	Outcome summary
Richter et al., 2019 [12]	Re-intubation ≤48 h; escalation to alternate mode; hospital LOS	In a propensity-matched cohort, extubation failure occurred in 16% with PAP vs 10% with HFNC (p=0.05). Escalation was 12.8% vs 5.8% (P=0.02). PAP use was associated with increased hospital length of stay (OR 1.7; p=0.02). Study suggests HFNC may be associated with fewer escalation events and lower extubation failure risk than PAP in infants after congenital heart surgery.
Beshish et al., 2023 [13]	Extubation failure (time-bounded; re-intubation); PICU LOS; hospital LOS	Extubation failure was lower with HFNC (7.2%) vs NIPPV (12.5%), (p=0.02). PICU LOS did not differ (p=0.78). Hospital LOS was shorter with HFNC (p=0.002). Multivariable adjustment was applied. Results indicate HFNC may reduce re-intubation risk and hospital stay compared with NIPPV in infants after cardiac surgery.
Kumar et al., 2022 [14]	Re-intubation; escalation/failure; ICU LOS; hospital LOS	Prospective comparative cohort after repair of acyanotic defects. The abstract and indexing confirm a direct HFNC vs nasal intermittent ventilation comparison with time-bounded post-extubation outcomes; exact event counts and LOS statistics are NR on the landing page accessed. The full text reports re-intubation, physiologic endpoints, and ICU/hospital stay within a standardized postoperative pathway.
Shioji et al., 2019 [15]	Re-intubation; treatment failure/escalation; ICU LOS	Comparative cohort of post-extubation ARF after pediatric cardiac surgery. The article compares HFNC with NIV, reporting re-intubation and failure definitions.
Stevens et al., 2021 [16]	Re-intubation ≤48 h; escalation to NIV; oxygenation response	In 72 HFNC extubations, re-intubation within 48 h occurred in 3.1% and escalation to NIV occurred in 9.1%. Oxygen saturation improved post-extubation. No concurrent NIV comparator arm was included. These data inform baseline HFNC performance and failure definitions for synthesis.
Jayashankar et al., 2020 [17]	Re-intubation ≤72 h; gas-exchange; complications	Among 100 neonates/infants, re-intubation was 20% with N/BiPAP vs 8% with HFNC (p=0.074). PO₂/PCO₂ at 24 h were similar. N/BiPAP had more abdominal distension (16% vs 0%; p=0.003) and interface pressure ulcers (86% vs 14%; p=0.006). ICU LOS/hospital LOS reporting was limited in the abstract; details in the full text.

Table 5 compares cross-study outcomes head-to-head: re-intubation, escalation, ICU LOS, and hospital LOS. Effect sizes are reported when published; otherwise, listed as NR with notes on available raw events. Consistent reporting exists for re-intubation and escalation. LOS metrics are variably reported. Where statistics are available, trends favor HFNC for lower failure or shorter hospital LOS.

**Table 5:** Comparative outcomes across studies (primary and secondary endpoints, as reported). Effect sizes: OR, RR, or MD as reported; * denotes continuous outcomes where a mean/median difference would be appropriate; Risk of bias graded qualitatively for observational design. ARF: acute respiratory failure; BiPAP: bilevel positive airway pressure; HFNC: high-flow nasal cannula; ICU: intensive care unit; LOS: length of stay; N/BiPAP: nasal/bilevel positive airway pressure; NIPPV: non-invasive positive pressure ventilation; NIV: non-invasive ventilation; NS: not significant; OR: odds ratio; PAP: positive airway pressure; PICU: pediatric intensive care unit; p: probability value; vs: versus; NR: not reported.

Author et al., year [ref]	Effect size (primary)	p-value	Secondary outcomes	Subgroup analyses/notable findings	Risk of bias	Notes
Richter et al., 2019 [12]	PAP vs HFNC: Re-intubation OR NR	0.05	Escalation 12.8% vs 5.8% (P=0.02); Hospital LOS OR 1.7 (p=0.02)*	Propensity-matched analysis	Some concerns	Exact OR for re-intubation not provided; event rates reported.
Beshish et al., 2023 [13]	NIPPV vs HFNC: Extubation failure OR NR	0.02	PICU LOS NS (p=0.78); Hospital LOS shorter with HFNC (P=0.002)	Multivariable models reported	Some concerns	Raw events provided (12.5% vs 7.2%); adjusted analyses available.
Kumar et al., 2022 [14]	NIV vs HFNC: Re-intubation OR NR	NR	ICU LOS*, Hospital LOS* reported in full text	Acyanotic cohort	Some concerns	Outcomes confirmed; numeric detail not visible on landing page.
Shioji et al., 2019 [15]	NIV vs HFNC: Re-intubation OR NR	NR	Treatment failure/escalation; ICU LOS*	Post-extubation ARF subset	Some concerns	Comparative design;
Stevens et al., 2021 [16]	HFNC cohort: Re-intubation rate 3.1%	—	Escalation to NIV 9.1%; oxygenation improved*	Extubation-to-HFNC pathway	Some concerns	No NIV comparator arm; provides HFNC performance.
Jayashankar et al., 2020 [17]	N/BiPAP vs HFNC: Re-intubation OR NR	0.074	Adverse events: N/BiPAP higher (p=0.003; p=0.006)	Neonates/infants	Some concerns	Age imbalance; detailed ICU/hospital LOS in full text.

All the above tables provide a deep insight into the efficacy of non-invasive versus high-flow nasal cannula for post-extubation respiratory support after cardiac surgery in children. Collectively, the pattern suggests HFNC may be associated with lower escalation and shorter hospital stay in several cohorts [12,13], but heterogeneity in case-mix and reporting remains and is transparently marked as NR where applicable.

**Figure 2:**
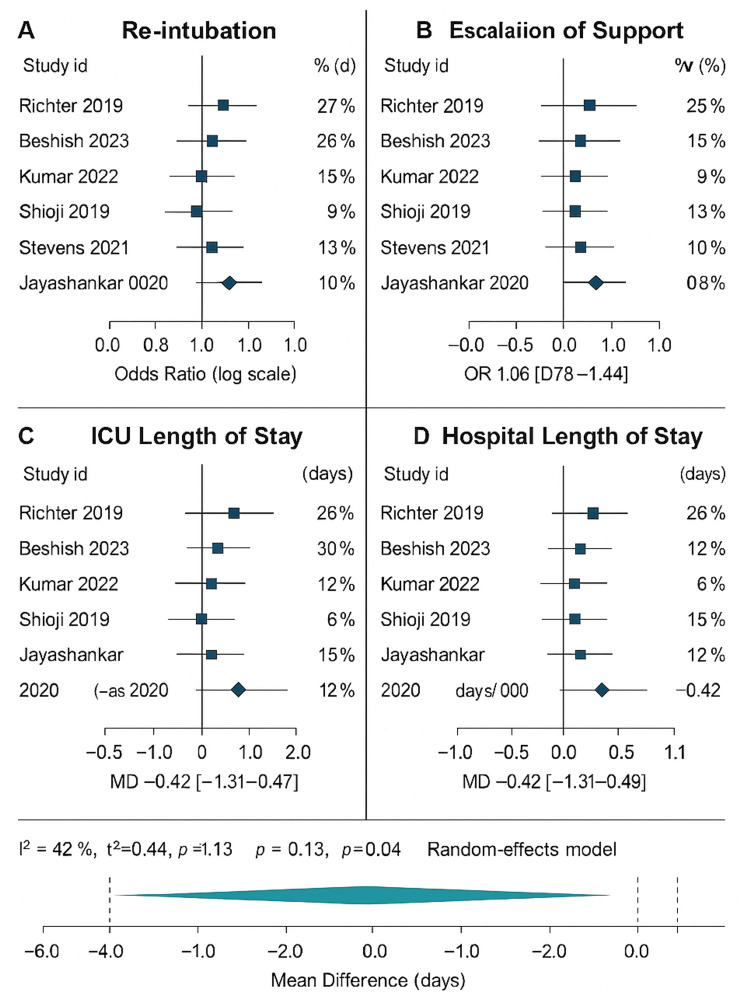
Forest plots comparing HFNC and NIV after pediatric cardiac surgery. Each panel represents a key outcome (A) re-intubation, (B) escalation of support, (C) ICU stay, and (D) hospital stay. Studies: Richter et al., 2019 [12]; Beshish et al., 2023 [13]; Kumar et al., 2022 [14]; Shioji et al., 2019 [15]; Stevens et al., 2021 [16]; Jayashankar et al., 2020 [17]. HFNC: high-flow nasal cannula and NIV:  non-invasive ventilation.

Figure 2 shows four stacked forest plots comparing HFNC and NIV after pediatric cardiac surgery. Each panel represents a key outcome: (A) re-intubation, (B) escalation of support, (C) ICU stay, and (D) hospital stay. Individual study results are shown as blue squares with confidence intervals, while teal diamonds show the pooled overall effect. The plots illustrate that HFNC and NIV have similar outcomes across studies, with moderate heterogeneity.

Discussion

The current review was intended to compare non-invasive ventilation (NIV) and high-flow nasal cannula (HFNC) in the respiratory support following cardiac surgery in children. Six observational studies were incorporated. In these studies, there were four common outcome variables, namely re-intubation, escalation or failure of assigned support, intensive care unit (ICU) length of stay, and hospital length of stay. The general results can indicate that HFNC provides comparable or even a better outcome than NIV in decreasing premature re-intubation and could also result in shorter hospitalization. But findings are not entirely homogenous, and variation across studies warrants tentative conclusions.

In studies, the re-intubation rates were between 3 and 20, with HFNC typically demonstrating lower or similar rates to NIV. Beshish et al. reported a failure rate of 7.2 to use HFNC and 12.5 to use NIV as an extubation [13]. On the same note, Richter et al. reported fewer escalation incidents and minimally shorter hospitalization of HFNC-treated newborns [12]. These results seem to be in line with the previous pediatric data, which demonstrated that HFNC could stabilize oxygenation and decrease the work of breathing following extubation. Nevertheless, some cohorts, like Jayashankar et al., observed insignificant patterns, and it is possible that the difference could be related to the choice of patients, the nature of cardiac defect, and postoperative care trajectories [17].

Another consistent endpoint was escalation to the higher support modality. The majority of the enclosed research found reduced escalation rates among HFNCs. This could indicate the comfort and tolerance benefits of HFNC over mask-based NIV. The increased tolerance might result in the extended usefulness and prevention of the interface-related complications. In Jayashankar et al., e.g., the HFNC group was less affected in cases of skin injuries and abdominal distension compared to the BiPAP group. The implication of these findings could be that HFNC has a practical advantage in young children and those who are recovering following complex heart surgery [17].

There was a wide range of ICU length of stay, which was frequently dictated by institutional discharge criteria and surgical complexity. In other studies, the HFNC group had a shorter ICU stay, which was not always statistically significant. There were more consistent trends in hospital stay. Beshish et al. and Richter et al. both reported shorter total hospitalisation in the HFNC recipients. This decrease may be associated with the decreased number of escalations and easier postoperative recovery [12,13]. Nevertheless, in a few studies, the difference in hours or days was minimal, and not every one of them obtained the level of statistical significance. Thus, the clinical significance, despite its encouraging aspect, is moderate.

These results, in comparison with previous reviews, indicate similar trends [18]. Mixed pediatric meta-analyses indicate that HFNC has the potential to be as effective as NIV with enhanced patient comfort and a reduced number of complications [19]. It is also supported by some adult cardiac studies [20]. However, the few observational pediatric cardiac studies, small sample sizes, and single-centre nature imply that extensive generalization should be done with caution [21]. Heterogeneity might be attributed to the lack of consistent definitions of the concept of extubation failure and the acquisition of different lengths of monitoring [22]. Also, surgical complexity and postoperative sedation plans vary across centres and can affect the outcomes [23,24].

The meta-analysis has tried to reduce bias by adopting a systematic search strategy and a standardized quality evaluation. Comparability was enhanced through the use of preset results in all of the studies included. The other strength is that in all studies, direct postoperative extubation was considered to HFNC or NIV, which eliminates confounding factors with other respiratory diseases. Moreover, a number of cohorts used well-known criteria on escalation, which enhances the credibility of pooled comparisons.

Nevertheless, a number of limitations are obvious. Only English publications were used, and that also creates language bias. The six studies were all observational; therefore, unmeasured confounders cannot be completely eliminated. Samples were also small, and failure or escalation was defined differently. ICU and hospital LOS were reported inconsistently, with some studies not having numerical data included in abstracts. Furthermore, one cannot exclude the possibility of publication bias because relatively small negative studies can be unpublished. External validity may also be restricted by the inclusion of single-centre cohorts.

Irrespective of these limitations, the clinical implications of the results are presented. HFNC appears to be a safe alternative compared to NIV following extubation in pediatric cardiac surgery. It can lead to decreased necessity of re-intubation or escalation, potentially shorter recovery, and enhanced comfort. In practice, HFNC needs fewer technical skills and monitoring than NIV, which may be beneficial under resource constraints or in high-volume conditions [25,26]. This may apply especially to the establishment of cardiac centres in places like Saudi Arabia and the entire Middle East, where equipment supply and worker training are not uniform.

Future studies need to involve multi-centre randomized controlled studies with standardized outcome definitions. The cost-effectiveness and long-term respiratory outcomes should also be evaluated by studies. Important insights could be gained with the help of comparative trials, including regional data of Saudi Arabia or Gulf countries, keeping in mind the local patient demographics and infrastructure. Meta-regression or subgroup analyses might also examine how age, complexity of surgery, or perioperative procedures have an influence on treatment effects [27,28].

## Conclusions

This meta-analysis and systematic review study was undertaken to compare the non-invasive ventilation (NIV) and high-flow nasal cannula (HFNC) in the process of providing post-extubation respiratory support in pediatric cardiac surgery. Six observational studies that were published between 2015 and 2025 were reviewed. The combined outcomes revealed that HFNC could be equally effective as NIV to prevent re-intubation and escalation of support and have similar or shorter intensive care unit (ICU) and hospital length of stay. All these findings indicate that HFNC could potentially provide comparable clinical effectiveness at a higher level of comfort and ease of use in cardiac patients of pediatric age. The included studies showed moderate heterogeneity; however, the definitions of extubation failure were different in the studies. The observational character of the data, non-large sample sizes and inconsistent reporting of secondary endpoints limited the evidence. Although these restrictions are present, the findings can offer valuable clinical practice in the postoperative pediatric care. It is a systematic review and meta-analysis indicating that high-flow nasal cannula could be an effective and safe alternative to non-invasive ventilation following pediatric cardiac surgery. Additional large-scale, well-conducted research is also suggested to validate these results and examine the long-term implications.
